# Applying Flora Composition and Leaf Physiognomy to Reconstruct the Paleocommunity, Palaeoclimate, and Paleoenvironment of the Jehol Biota in Jilin, China

**DOI:** 10.3390/plants15010022

**Published:** 2025-12-20

**Authors:** Wei Huang, Dejun Zhang

**Affiliations:** 1 College of Life Sciences, Jilin Normal University, No. 1301 Haifeng Street, Siping 136000, China; 18543142408@163.com; 2 Shenyang Center of Geological Survey, China Geological Survey, Shenyang 110034, China; 3 MNR Key Laboratory of Stratigraphy and Palaeontology, Institute of Geology, Chinese Academy of Geological Sciences, Beijing 100037, China

**Keywords:** Baishan city, Early Cretaceous, Jehol Biota, paleoecology, paleoclimate

## Abstract

Herein, we present a systematic investigation of plant fossils from the Yingzuilazi Formation in Baishan City, Jilin Province, China. The Baishan flora comprises 27 genera and 46 species. They are predominantly autochthonous or parautochthonous, based on their floral composition and taphonomic attributes. An analysis of paleoecological characteristics of the fossil plant assemblages, combined with the habitat preferences of analogous modern communities, allowed us to reconstruct the Early Cretaceous plant communities in the Baishan Basin: a riparian–wetland community, lowland community, montane slope community, and montane highland community. The floral composition, a statistical analysis of foliar physiognomy, and the palynofloral characteristics indicated a warm and humid temperate climate during the deposition of the Yingzuilazi Formation. A genus-level comparison with the Yixian Formation flora of western Liaoning revealed high compositional similarity, which confirms the Baishan flora as the easternmost distribution of the Jehol Biota in China. This study provides new fossil evidence for understanding Early Cretaceous floristic provincialism and paleoenvironmental reconstruction in East Asia. It offers geological references that can predict vegetation responses to a greenhouse climate. Additionally, Sphenopsida and Filicopsida may serve as potential indicators that may identify favorable terrestrial shale oil and gas reservoirs from the Early Cretaceous.

## Simple Summary

This study addresses the limited understanding of Early Cretaceous paleoflora in the Baishan Basin, Jilin Province, China. The research aimed to systematically analyze newly discovered plant fossils from the Yingzuilazi Formation to reconstruct the paleocommunity and paleoclimate. The findings identified 46 plant species, which were predominantly autochthonous or parautochthonous. The analysis allowed for the reconstruction of four distinct plant communities. Their floral composition indicated that the region experienced a warm and humid temperate climate during the Early Cretaceous. Furthermore, the high similarity with the famous Jehol Biota from western Liaoning confirmed the Baishan flora as its easternmost known distribution. These conclusions are significant for multiple reasons. They provide crucial data for understanding the Early Cretaceous floristic provincialism and paleoenvironmental reconstruction in East Asia. The study offers a geological benchmark for predicting how modern vegetation might respond to a future greenhouse climate. Finally, certain plant groups identified (Sphenopsida and Filicopsida) may serve as useful indicators for exploring the terrestrial shale oil and gas reservoirs from this period.

## 1. Introduction

The Jehol Biota, which represents one of the most exceptionally preserved Early Cretaceous terrestrial ecosystems, has been studied for nearly a century. The initial studies suggested that its primary distribution comprises northern Hebei, western Liaoning, and southeastern Inner Mongolia in China. During the last four decades, a wealth of exquisitely preserved and taxonomically diverse fossils have yielded about the Jehol Biota [1,2,3,4,5,6,7,8,9,10], revealing a significantly expanded geographic range that includes the Korean Peninsula, Japan, Mongolia, Kazakhstan, and Siberia [11,12]. The initial studies of the Jehol Biota centered on phylogeny of individual clades or application of single disciplines (paleontology, geochemistry, geochronology) [13]. The research has now evolved into integrated investigations into the Jehol Biota’s origins, evolution, and extinction, and its interactions with coeval paleoclimatic fluctuations and paleoenvironmental changes [4,14,15,16,17,18,19,20]. In summary, the Jehol Biota has become a research focus in paleobiology and evolutionary biology.

The Jehol Biota encompassed nearly all major plant divisions, even the phylogenetically significant, though non-dominant, early angiosperms—a group that potentially originated in aquatic environments [21,22,23,24,25]. Nevertheless, a critical knowledge gap persists regarding the floristic diversity composition and paleoecology of the marginal distribution zones of the Jehol Biota, significantly constraining our understanding of its spatial expansion mechanisms and climatic adaptation strategies.

Abundant fossil plants, fauna, and palynomorphs have been obtained from the same area of the Yingzuilazi Formation in the Baishan Basin, Jilin Province [26,27]. The identified fauna includes invertebrates, such as Conchostraca, Ostracoda, Gastropoda, Bivalvia, and Insecta, as well as vertebrates, including fish and caudate amphibians. The Conchostraca fossils are primarily represented by *Eosestheria*, a representative element of the Jehol Biota. The Ostracoda include the following: *Cypridea* (*Cypridea*) *baishanensis* Zhang, *Cypridea* (*Cypridea*) *tubercularis* Pang, *Cypridea* (*Cypridea*) *vitimensis* Mandelstam, *Cypridea* (*Cypridea*) sp., *Cypridea* sp.1, *Cypridea* sp.2, *Cypridea* sp.3, *Lycopterocypris infantilis* Lübimova, *Damonella* sp., *Yumenia heitizigouensis* Zhang, and *Darwinula contracta* Mandelstam. Many of these taxa are also commonly found in the Yixian Formation of western Liaoning. The bivalve fossils are relatively limited in diversity, mainly consisting of *Arguniella* sp., *Arguniella subcentralis* Chernyshev et Gu, and *Arguniella lingyuanensis* Gu. Gastropod fossils are relatively scarce in the Yingzuilazi Formation, represented only by *Probaicalia vitimensis* Martinson and *Probaicalia* sp. This genus is also a frequent component of the Jehol Biota in the Yixian Formation of western Liaoning. The insect fossils are represented by *Ephemeropsis trisetalis* Eichwald and *Sinochresmoda magnicornia* Zhang, Ren et Pang. Although the fish fossils are fragmentary, the size and morphology of the skeletal elements are consistent with those of *Lycoptera*, a representative fish genus of the Jehol Biota. The caudate amphibian fossil is well preserved and can be attributed to salamanders.

The *Eosestheria*–*Ephemeropsis trisetalis*–*Lycoptera* (EEL) assemblage has long been recognized as a defining component of the Jehol Biota and is highly significant for determining the stratigraphic age and delineating the biogeographic distribution of the biota. The presence of *Lycoptera* sp., *Ephemeropsis trisetalis* Eichwald, and *Eosestheria* spp. in the present assemblage firmly establishes a strong correlation with the Jehol Biota; coupled with the established biostratigraphic correlations, it confirms that Baishan City is the easternmost biogeographic extent of the Jehol Biota in China [28]. But the compositional features, community structure, and paleoclimatic significance of the Baishan flora remain systematically underexplored. The preliminary data have revealed significant differences in the proportions of gymnosperm groups (Coniferales, Czekanowskiales) between the Baishan flora and the Yixian Formation flora of western Liaoning [29]. This suggests that the phytogeographic patterns in East Asia may have been driven by regional topographic and climatic heterogeneity during the Early Cretaceous.

This study systematically analyzes the plant fossil assemblage from the Baishan Basin. It focuses on investigating the paleoecology, which involves reconstructing the paleocommunity, inferring the paleoenvironment and paleoclimate. This work provides new paleontological constraints for refining models of seasonal temperate climates in East Asia during the Early Cretaceous.

## 2. Materials and Methods

**Figure 1:**
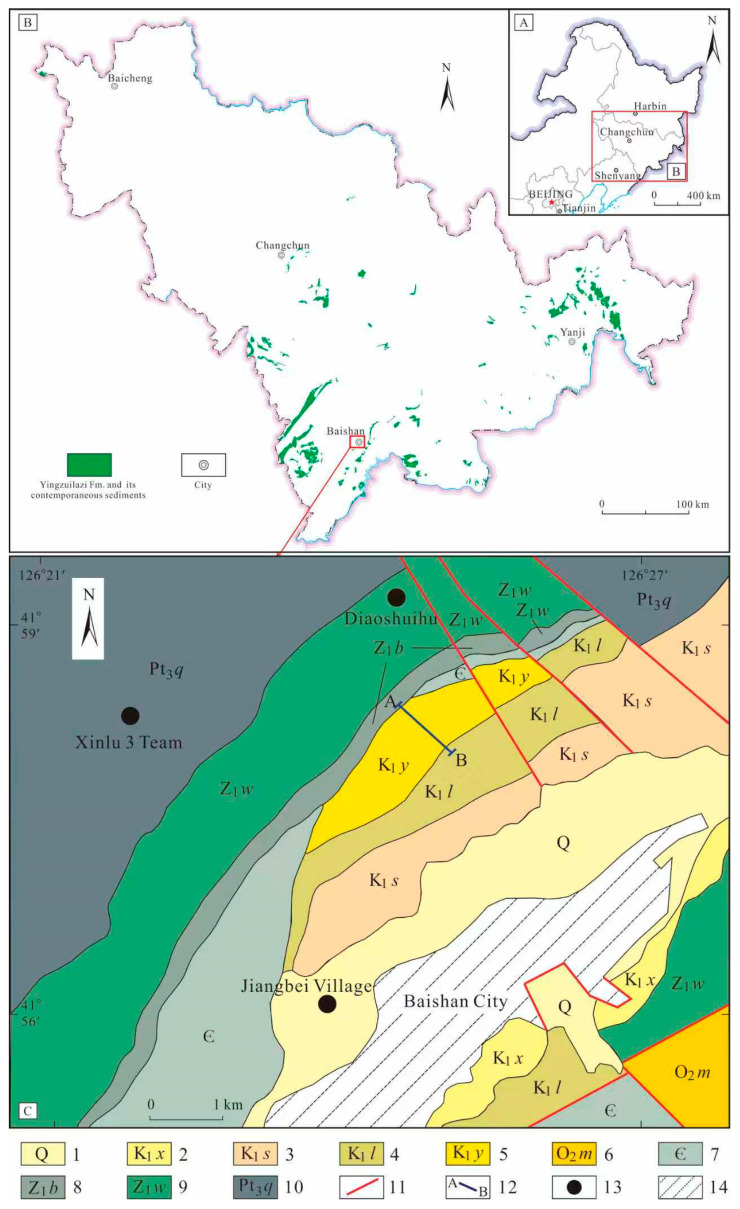
Location of the Study Area. (**A**). The geographic location of fossil site in Northeast China, (**B**). Stratigraphic Distribution of the Early Cretaceous in Jilin Province, (**C**). Sampling Sections and Regional Geology of the Baishan Area. 1. Quaternary System. 2. Lower Cretaceous Xiaonangou Formation. 3. Lower Cretaceous Shiren Formation. 4. Lower Cretaceous Linzi tou Formation. 5. Lower Cretaceous Yingzuilazi Formation. 6. Middle Ordovician Majiagou Formation. 7. Cambrian System. 8. Tonian Badaojiang Formation. 9. Tonian Wanlong Formation. 10. Qingbaikouan System (Upper Proterozoic). 11. Fault. 12. Stratigraphic Section. 13. Village. 14. City.

The Baishan Basin is an Early Cretaceous small-scale faulted continental sedimentary basin. Tectonically, it occupies the northeastern margin of the North China Craton, bounded by the Tan–Lu Fault Zone to the east and the Tianshan–Xingmeng Orogenic Belt to the north. Its southern margin adjoins the Liaodong Block, with eastward extensions connecting it to the Korean Peninsula basement [13,30]. The Late Mesozoic stratigraphy belongs to the Coastal Pacific Stratigraphic Domain, the Southern Jilin–Eastern Liaoning Stratigraphic Subregion, and the Tonghua Stratigraphic Microregion in the Baishan Basin (Jilin Bureau of Geology and Mineral Resources). The stratigraphic sequence comprises, in ascending order, the following: Lower Cretaceous Yingzuilazi Formation (K_1_*y*), Linzitou Formation (K_1_*l*), Shiren Formation (K_1_*s*), and Xiaonangou Formation (K_1_*x*) (Figure 1).

**Figure 2:**
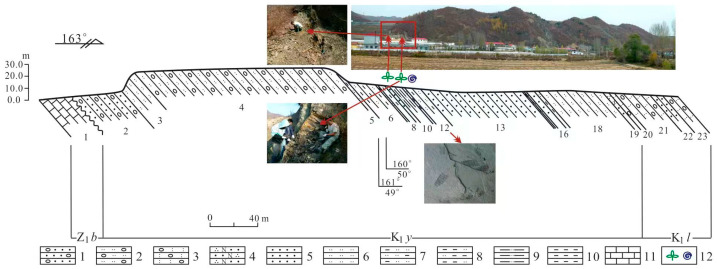
Measured section of the Lower Cretaceous Yingzuilazi Formation in the Baishan Basin, southeastern Jilin Province. 1. Gravel-bearing sandstone. 2. Gravel-bearing siltstone. 3. Tuffaceous conglomerate. 4. Feldspathic quartz sandstone. 5. Fine sandstone. 6. Siltstone. 7. Argillaceous siltstone. 8. Silty mudstone. 9. Silty shale. 10. Mudstone. 11. Limestone. 12. Fossil-bearing horizon (fauna and plant fossils).

The Yingzuilazi Formation unconformably overlies the Sinian Badaojiang Formation and underlies the Linzitou Formation. It consists of fluvial–lacustrine siliciclastic deposits, including conglomerates, sandstones, siltstones, mudstones, and coal streaks, yielding diverse animal, plant, and palynomorph fossils. The measured section (base coordinates: 41°58′37.5″ N, 126°25′39.9″ E) exhibits an unconformable contact with the conglomerate underlying the Sinian Badaojiang Formation and a conformable upper contact with the tuffaceous conglomerates of the Linzitou Formation, with a total thickness of 363.7 m (Figure 2).

The fossils were examined using an Olympus SZX series stereomicroscope (model:BX51,manufacturer:Olympus Corporation, Japan)to assess their morphological features, which were then documented, described, and preliminarily classified. The specimens were photographed with a digital camera (model:Canon 5D Mark II, manufacturer:Canon Inc., Japan). For the plant compression fossils, a cuticular analysis was performed. In cases of fossils with poorly preserved cuticles or preserved as impressions, observations were carried out using both stereomicroscopy and a 3D digital microscope (model:VHX-1000,manufacturer:KEYENCE Corporation, Japan) to attempt to resolve the residual microscopic features. These features were partially reconstructed with computer software (CorelDRAW Technical Suite 2023). For fossil leaves from which cuticles could be obtained, epidermal macerations were prepared, and samples were collected for Scanning Electron Microscopy (model:JSM-6700F, manufacturer:JEOL Ltd., Japan). These samples were then observed and photographed using a light microscope (model:VHX-1000,manufacturer:KEYENCE Corporation, Japan) and an SEM, respectively. The palynological data cited in this study are derived from previously published research. Due to the age of that publication and limited access to the original materials, this study cannot provide updated high-resolution images or further details regarding slide preparation. The cited palynological information is used only as a supplementary reference material to support the regional paleoenvironmental context. We have clearly indicated its source and limitations to avoid overinterpretation of the original data. All the morphological characteristics and taxonomic determinations are based on the macrofossil specimens collected, identified, and thoroughly described in the present study, with the associated images and measurements fully provided in the manuscript.

From an analysis of the lithology, sedimentary record, and distribution of fossils from the measured section of the Yingzuilazi Formation, the following paleoenvironment is reconstructed. The lower part of the formation consists of thick-bedded purplish conglomerates intercalated with medium-bedded siltstones. The conglomerates, with clasts mostly 2–5 cm in size, are matrix-supported and moderately rounded, suggesting deposition in a fluvial channel bar or lakeshore environment. This purplish conglomerate suite may reflect relatively arid climatic conditions during the initial depositional stage of the Yingzuilazi Formation. The middle part shows a noticeable fining in grain size, dominated by gray, yellow, or yellowish-green medium- to thin-bedded sandstones, siltstones, and mudstones, with well-developed horizontal bedding. This interval contains fossils, such as conchostracans, ostracods, fish, and bivalves, indicating deposition in a shallow lacustrine environment. The upper part is mainly composed of gray and yellow medium- to thick-bedded sandstones interbedded with siltstones and mudstones. Bivalve fossils are dominant in this section, occurring densely and in distinct layers, representing a gentle shallow lakeshore depositional setting (Table 1).

All the fossil specimens analyzed in this study were collected from the Yingzuilazi Formation in Baishan City, Jilin Province, China, and are preserved at the Paleontological Research Center of Jilin University.

### 2.1. Taxonomic Composition of Baishan Flora

#### 2.1.1. Taxonomic Composition

The Yingzuilazi Formation macroflora comprises 27 genera and 46 species (Table 2), including Sphenopsida, Filicopsida, Cycadopsida, Ginkgoopsida, Czekanowskiales, Coniferopsida, Gnetophyta, reproductive organs, and seeds of gymnosperms.

The Bashan flora is dominated by gymnosperms and ferns. Coniferopsida exhibits the highest diversity, with 8 genera and 14 species, representing 30.43% of total species. Czekanowskiales account for 26.09% of this flora, and rank second with 5 genera and 12 species. These are followed by Ginkgoopsida (4 genera, 5 species; 10.87%) and gymnosperm reproductive structures (2 genera, 5 species; 10.87%). Filicopsida occupies the fifth position at 8.7%, which includes 3 genera and 4 species.

Other plant fossil groups are rare, including Sphenopsida (2 genera, 3 species; 6.52%) and Gnetophyta (2 genera, 2 species; 4.35%); Cyadopsida has poor distribution, with only 1 genus and 1 species (2.17%).

#### 2.1.2. Characteristics of the Baishan Flora

**Figure 3:**
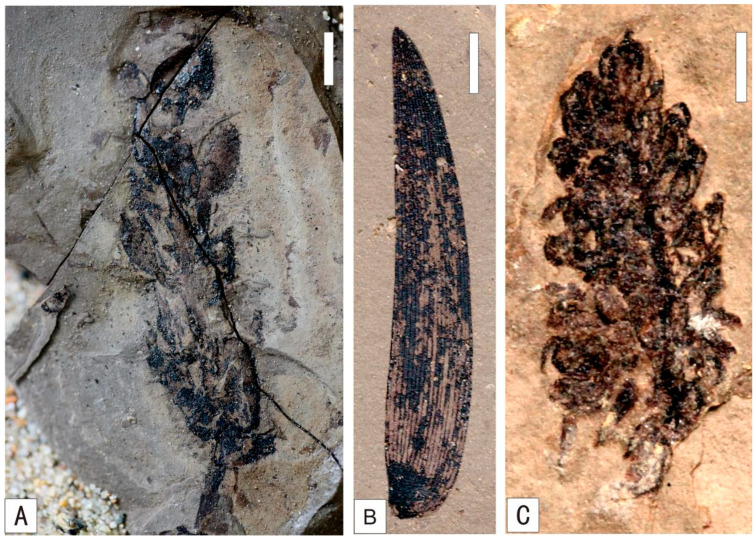
Early Cretaceous Coniferopsida in Baishan, Jilin Province. (**A**) *Schizolepis moelleri* Seward, (**B**) *Ferganiella podozamioides* Lih, (**C**) *Scarburgia hillii* Harris. (**A**,**B**) Scale = 5 mm. (**C**) Scale = 2 mm.

Coniferopsida exhibit the highest diversity in the Baishan flora and hold absolute dominance. As primary components of temperate forests, Coniferopsida possess the richest generic and specific diversity among extant gymnosperms. Evolution of Coniferopsida entered a highly active phase in the Early Mesozoic, reaching its peak stage of evolution in the Late Jurassic or Early Cretaceous. Most of the genera and species in the Baishan flora represent the main constituents of northern Early Cretaceous floras. Although *Pityophyllum* are not abundant, they display a relatively high diversity and serve as a diagnostic taxon of Bashan flora. The principal representatives of *Pityophyllum* include *Pityophyllum staratschini* (Heer) Nathorst and *Pityophyllum lindstroemi* Nathorst, indicating its relative flourishing during the depositional period. *Schizolepis* is represented by *Schizolepis moelleri* Seward (Figure 3A), while *Scarburgia* occurs as *Scarburgia hillii* Harris (Figure 3C). *Ferganiella* is documented as *Ferganiella podozamioides* Lih (Figure 3B). *Pityospermum*, *Pityocladus*, *Ferganiella*, *Lindleycladus*, and *Podozamites* contain multiple indeterminate species requiring further investigation.

**Figure 4:**
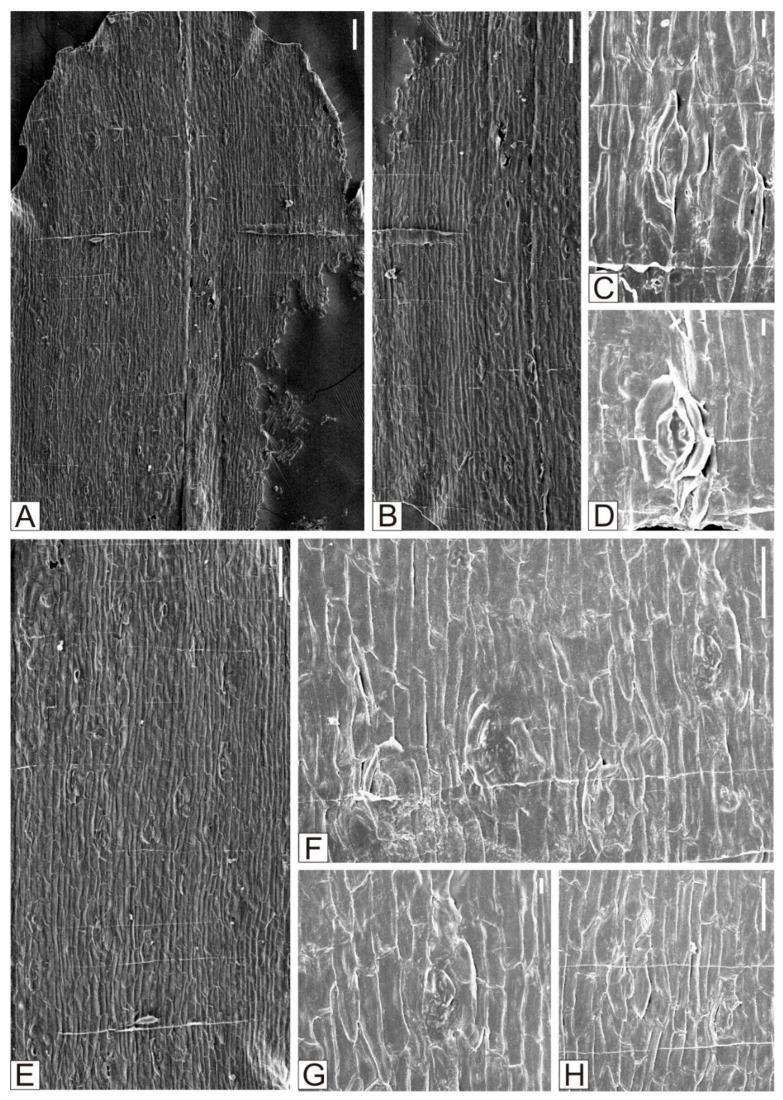
*Solenites murrayana* Lindley et Hutton. (**A**) The inner surface of the upper epidermis and lower epidermis of *Solenites murrayana* Lindley et Hutton. (**B**–**D**) The inner surface of the upper epidermis of *Solenites murrayana* Lindley et Hutton, and the morphology of the stomatal apparatus. (**E**–**H**) The inner surface of the lower epidermis of *Solenites murrayana* Lindley et Hutton, and the morphology of the stomatal apparatus. (**A**,**B**,**E**). Scale = 100 μm. (**C**,**D**,**G**) Scale = 10 μm. (**F**,**H**) Scale = 50 μm.

**Figure 5:**
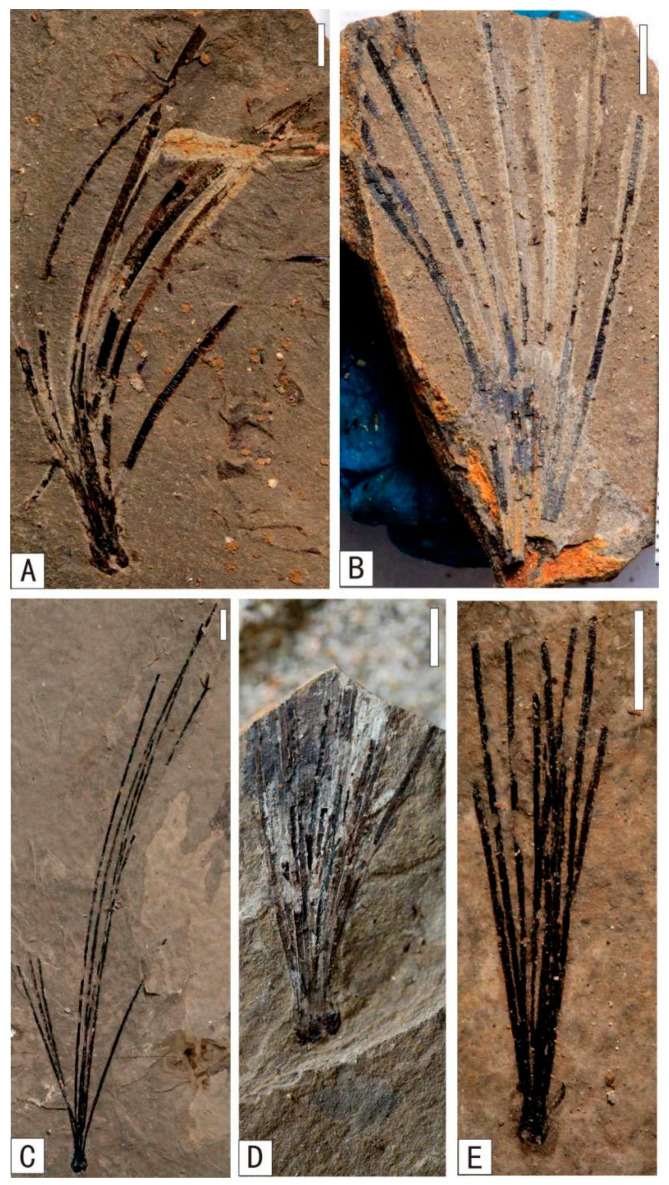
Early Cretaceous genus *Solenites* in Baishan, Jilin Province. (**A**) *Solenites baishanensis* Li et Sun, (**B**) *Solenites murrayana* Lindley et Hutton, (**C**) *Solenites gracilis* Li et Sun, (**D**) *Solenites* sp.2, (**E**) *Solenites* sp.1. (**A**–**E**) Scale = 5 mm.

**Figure 6:**
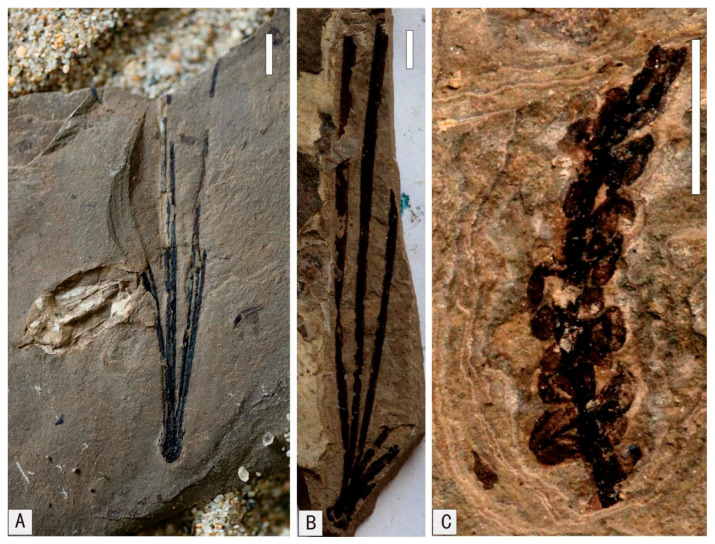
Early Cretaceous *Czekanowskiales* in Baishan, Jilin Province. (**A**) *Czekanowskia setacea* Heer, (**B**) *Phoenicopsis* (*Culgoweria*) *uralensis* Kiritchkova, (**C**) *Ixostrobus delicatus* Sun et Zheng. (**A**–**C**) Scale = 5 mm.

Czekanowskiales flourish as the second predominant group in the Bashan flora, comprising 5 genera and 12 species. Czekanowskiales are one of the most common fossil plant groups across Mesozoic Eurasia. Some genera in the present flora, such as *Czekanowskia*, *Phoenicopsis*, *Solenites*, and *Sphenarion*, are all common elements in Mesozoic floras. Among them, *Solenites* exhibits slightly higher diversity and abundance, with its representative members including *Solenites baishanensis* Li et Sun, *Solenites murrayana* Lindley et Hutton (Figure 4), *Solenites gracilis* Li et Sun, *Solenites* sp.1, and *Solenites* sp.2 (Figure 5). *Solenites* is equally prevalent in the Lower Cretaceous Yixian Formation. Other taxa of Czekanowskiales, such as *Phoenicopsis, Czekanowskia,* and *Ixostrobus*, are also frequently found in the Yixian Formation. These genera have also been discovered in the Bashan flora, with representative species like *Phoenicopsis* (*Culgoweria*) *uralensis* Kiritchkova, *Czekanowskia setacea* Heer, and *Ixostrobus delicatus* Sun et Zheng (Figure 6).

**Figure 7:**
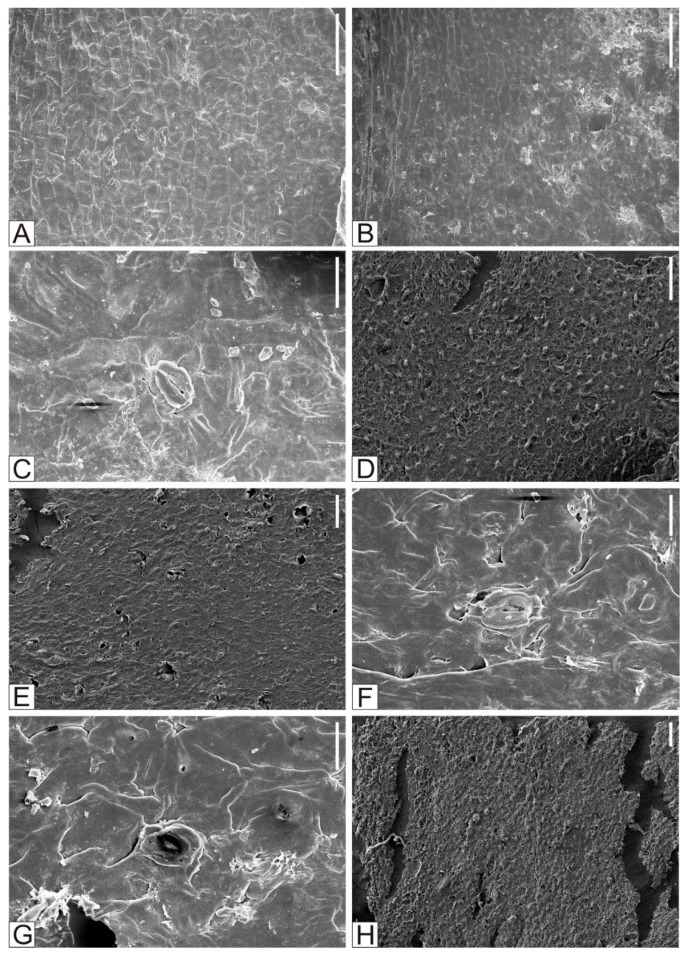
*Ginkgo huttoni* (Sternberg) Heer. (**A**–**C**) The inner surface of the upper epidermis of *Ginkgo huttoni* (Sternberg) Heer. (**D**) The outer surface of the upper epidermis of *Ginkgo huttoni* (Sternberg) Heer. (**E**–**G**) The inner surface of the lower epidermis of *Ginkgo huttoni* (Sternberg) Heer. (**H**) The outer surface of the lower epidermis of *Ginkgo huttoni* (Sternberg) Heer. (**A**,**B**,**D**,**E**,**H**) Scale = 100 μm. (**C**) Scale = 30 μm. (**F**,**G**) Scale = 20 μm.

**Figure 8:**
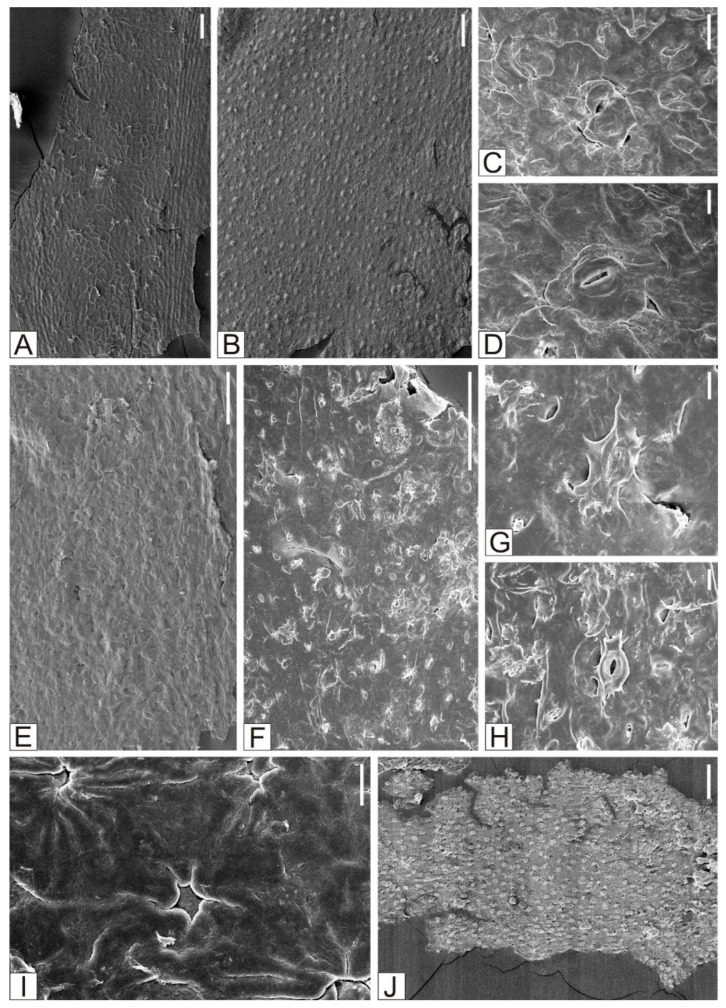
*Ginkgoites sibiricus* (Heer) Seward. (**A**) The inner surface of the upper epidermis of *Ginkgoites sibiricus* (Heer) Seward. (**B**) The outer surface of the upper epidermis of *Ginkgoites sibiricus* (Heer) Seward. (**C**–**I**) The inner surface of the lower epidermis of *Ginkgoites sibiricus* (Heer) Seward. (**J**) The outer surface of the lower epidermis of *Ginkgoites sibiricus* (Heer) Seward. (**A**,**B**,**E**,**F**,**J**) Scale = 100 μm. (**C**) Scale = 20 μm. (**D**,**G**,**H**,**I**) Scale = 10 μm.

**Figure 9:**
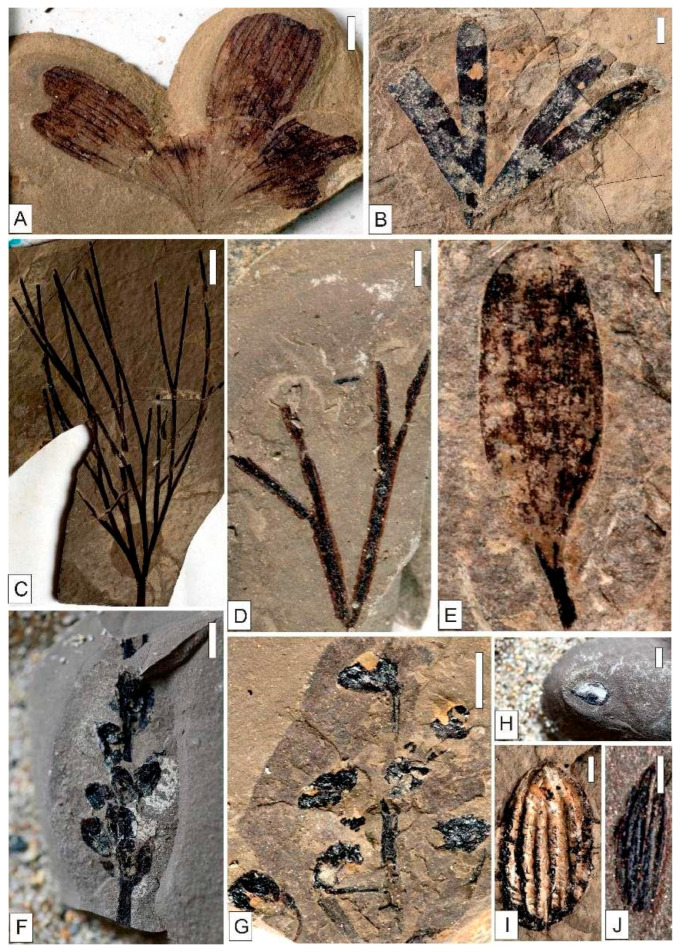
Early Cretaceous Ginkgoopsida, Fructus et Semina in Baishan, Jilin Province. (**A**) *Ginkgo huttoni* (*Sternberg*) Heer. (**B**) *Ginkgoites sibiricus* (Heer) Seward. (**C**) *Baiera baishanensis* Zhao et Sun. (**D**) *Baiera* sp. (**E**) *Pseudotorellia* sp. (**F**) *Strobilites* sp.1. (**G**) *Strobilites* sp.2. (**H**) *Carpolithus* spp. (**I**) *Carpolithus* sp.2. (**J**) *Carpolithus* sp.1. (**A**,**B**,**F**–**H**) Scale = 4 mm. (**D**,**E**,**I**,**J**) Scale = 2 mm. (**C**) Scale = 10 mm.

Ginkgoopsida and gymnosperm reproductive organs and seeds rank third among the Baishan flora, represented by 4 genera with 5 species and 2 genera with 5 species, respectively. Ginkgoopsida comprises 4 genera commonly found in Mesozoic flora: *Ginkgo*, *Ginkgoites*, *Baiera*, and *Pseudotorellia*; all exhibit significant morphological diversity in the extant flora. The principal representatives include *Ginkgo huttoni* (*Sternberg*) Heer (Figure 7), *Ginkgoites sibiricus* (Heer) Seward (Figure 8), *Pseudotorellia* sp., *Baiera baishanensis* Zhao et Sun, and *Baiera* sp. The fossil gymnosperm reproductive structures and seeds in the Baishan flora consist of *Strobilites* sp.1, *Strobilites* sp.2, *Carpolithus* sp.1, *Carpolithus* sp.2, and *Carpolithus* spp. (Figure 9).

Filicopsida is moderately represented in the Baishan flora, with 3 genera and 4 species. The group includes *Todites*, *Coniopteris*, and *Gleichenites*, which all show low diversity.

Sphenopsida, showing low diversity, contains 2 genera and 3 species, which are *Equisetites* and *Equisetostachys*. The representative members are *Equisetites* cf. *exiliformis* Sun et Zheng and *Equisetites longevaginatus* Wu, both showing slender-stemmed forms. They differ markedly from the thicker-stemmed forms of the Early–Middle Jurassic. Furthermore, the elements of Sphenopsida in the Baishan flora are similar to or identical with the holotypes of the Yixian Formation, and further support the depositional age of the Baishan flora.

Notably, *Ephedra* and *Ephedrites* are common in the Yixian Formation, which are present as *Ephedra* sp. and *Ephedrites* sp. in the Baishan flora.

**Figure 10:**
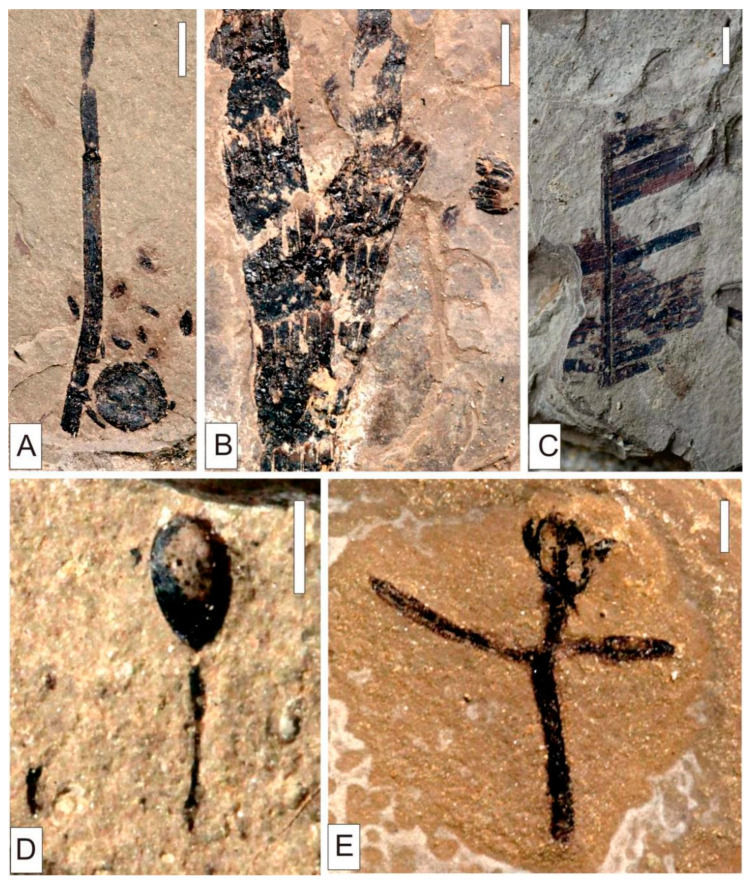
Early Cretaceous Sphenopsida, Cycadopsida in Baishan, Jilin Province. (**A**) *Equisetites* cf. *exiliformis* Sun et Zheng. (**B**) *Equisetites longevaginatus* Wu. (**C**) *Tyrmia* cf. *acrodonta* Wu. (**D**) *Ephedra* sp. (**E**) *Ephedrites* sp. (**A**,**B**) Scale = 4 mm. (**D**,**E**) Scale = 2 mm. (**C**) Scale = 5 mm.

Cycadopsida is extremely rare, represented by the single species *Tyrmia* cf. *acrodonta* Wu (Figure 10).

### 2.2. Paleoecology of the Flora

#### 2.2.1. Taphonomic Analysis

The critical step in reconstructing paleocommunities is analysis of taphonomy, which plays a vital role in restoring the original composition of fossil assemblages and their habitats [31]. An analysis of plant fossil preservation contributes to understanding plant life habits, paleoenvironmental restoration, and paleocommunity reconstruction. Bury types of fossils include autochthonous, parautochthonous, and allochthonous. Fine-grained host rocks typically indicate autochthonous or parautochthonous, while coarse-grained host rocks suggest allochthonous [32].

The lower section of Yingzuilazi Formation in the Baishan Basin is purple thick-bedded conglomerates intercalated with medium-bedded siltstones. The conglomerate clasts (mostly 2–5 cm) display good rounding and a matrix-supported fabric, representing fluvial channel bars or lacustrine shore deposits. This unit may reflect arid conditions during the initial deposition. The middle of the unit transitions to a finer grain, which is composed of gray, yellow, and yellowish-green sandstones, siltstones, and mudstones with well-developed horizontal bedding. Among them, fossils of plants, conchostracans, ostracods, bivalves, and fish indicate a shallow lake environment. The upper unit consists of gray–yellow medium-thick sandstones interbedded with siltstones and mudstones, containing densely layered bivalve fossils characteristic of low-gradient nearshore lacustrine settings.

Plant fossils occur exclusively in the fine-grained host rocks, suggesting autochthonous or parautochthonous preservation. Additional taphonomic criteria were evaluated, including fossil abrasion, sorting, stratigraphic position, and organ durability (Table 3). The comprehensive analysis of the Baishan flora’s preservation conditions, with results, is shown in Table 4. The richness is indicated by a ‘+’ for fewer fossils, ‘++’ for more abundant fossils, and ‘+++’ for rich fossils [33].

These findings demonstrate that the Baishan flora was preserved primarily through autochthonous and parautochthonous processes, providing an ecological representation of the original vegetation.

#### 2.2.2. Paleoecological Analysis of Plant Fossil Taxa

The paleobotanical and neontological research paradigms exhibit methodological continuity in the organ morpho-functional analysis. Morphological features of vegetative and reproductive organs reflect evolutionary adaptive responses to the environment. The incompleteness of plant fossil preservation in geological time commonly results in disarticulated organ assemblages. Reconstructing paleocommunity ecological structures is based on integrated morpho-anatomical studies of fossil organs with comparative analyses of niche parameters in extant relatives. This approach further requires inferences from paleogeographic patterns and paleoclimatic parameters. This research paradigm, spanning multiple time scales, not only advances our understanding of plant organ evolution but also establishes reliable biological proxy systems for paleoenvironmental reconstruction.

1. Sphenopsida

The extant Equisetales comprise a single family and genus with approximately 20 herbaceous species. These species exhibit significant height differentiation, stem diameters of 1–3 cm, and bear whorled vegetative or reproductive branches, all characterized by a slender morphology. The sphenopsid vegetative organs *Equisetites* cf. *exiliformis* Sun et Zheng and *Equisetites longevaginatus* Wu, and the reproductive organ *Equisetostachys* sp. have been found in Baishan flora. Morphological comparisons reveal consistent phenotypic traits between Mesozoic Equisetales and their extant counterparts. This similarity suggests analogous ecological habits as helophyte–hydrophyte communities, inhabiting river–lake margins, swamps, or directly in aquatic environments.

2. Filicopsida

Filicopsida represent pioneering clades in early evolution of vascular plants, maintaining ecological prominence in terrestrial floras since the Late Paleozoic. The extant taxa predominantly inhabit humid tropical to temperate regions, with peak abundance in tropical and subtropical zones. The extant Dicksoniaceae comprise 3 genera distributed across tropical, subtropical, and temperate zones of both hemispheres, often exhibiting tree–fern growth forms that indicate warm–humid climates and well-drained substrates. Gleicheniaceae (*Gleichenia*) and Osmundaceae (*Osmunda*) thrive in tropical to subtropical regions, favoring warm–moist environments or gentle mountain slopes. Filicopsida of the Baishan biota includes *Coniopteris angustiloba* Brick and *Coniopteris* sp. of Dicksoniaceae, *Todites* sp. of Osmundaceae, and *Gleichenites* sp. of Gleicheniaceae. Their frond morphology and structural features closely resemble those of their extant relatives, collectively indicating warm–temperate humid conditions with abundant water resources.

3. Cycadopsida

The fossils and extant cycads exhibit convergent foliar morphology, characterized by coriaceous pinnae ranging from centimeters to one meter in length, slightly thick cuticles, and commonly exhibit once-pinnate compound leaves. The paleoecological analyses indicate Cycadopsida’s primary adaptation to tropical and subtropical climates, with occasional occurrences in Mesozoic temperate regions [34].

Cycadopsida, a dominant Mesozoic plant group, comprises the extant Cycadales and extinct Nilssoniales and Bennettitales [35,36]. The modern Cycadales include 3 families, 11 genera, and approximately 305 species. Zamiaceae and Stangeriaceae predominantly occur in the tropical and subtropical southeastern USA, South America, Central America, Australia, and Africa [37]. Cycadaceae are concentrated in Asia, and tropical and subtropical Australia and Africa.

The Mesozoic cycads are segregated into two ecotypes. There are the humid tropical and subtropical taxa with fasciculate pinnae, tuberous, or unbranched barrel-shaped stems. The arid-adapted taxa combine slender stems and fasciculate foliage. Bennettitales share morpho-functional traits with Cycadales, suggesting their adaptation to warm–humid temperate–tropical zones [38,39,40]. Cycad fossils also occur in terrestrial interbeds of marine basins, highlands, and deltas [38,39,40,41]. The reported studies have identified Bennettitales genera *Zamites*, *Sphenozamites*, *Otozamites*, *Dictyozamites*, *Pseudocycas*, and *Ptilophyllum* as indicators of tropical and subtropical climates. *Pterophyllum*, *Amonozamites*, and *Nilssoniopteris* represent warm–humid tropical–subtropical or temperate–warm–temperate zones. The Baishan flora contains only one species that occurs in Siberian floras, *Tyrmia* cf. *acrodonta* Wu, closely resembling the holotype specimens from the Yixian Formation in western Liaoning.

4. Ginkgoopsida

Ginkgoopsida constitute the dominant components of temperate to warm–temperate vegetation, with their earliest fossil record extending to the Permian, predominantly preserved as foliar remains. Studies of their reproductive organs confirm the phylogenetic continuity between the fossils and extant Ginkgoales [42]. Ginkgo *biloba* Linne represents the sole extant species that is a deciduous tree favoring cool conditions, typically inhabiting warm–temperate or subtropical highlands and mixed conifer–broadleaf forests in temperate zones [43].

Fossils of Ginkgoopsida leaves are commonly palmately dissected. *Baiera* is distinguished from *Ginkgo* and *Ginkgoites* by the lobe dissection frequency and size. *Sphenobaiera* shares morphological and cuticular similarities with *Ginkgo* and *Baiera*, with the sole distinction being its sessile leaves. These patterns support the hypothesis of multiple scholars that Mesozoic *Ginkgo* (*Ginkgoites*) occupied habitat conditions similar to or equivalent with those of extant *Ginkgo* (*Ginkgoites*). Likewise, the occurrence of *Ginkgo*, *Ginkgoites*, *Baiera*, and *Pseudotorellia* in the Baishan flora reinforces the evidence that Mesozoic Ginkgoopsida thrived in warm–humid temperate montane forests and cool upland settings [23,44,45,46,47,48,49,50].

5. Czekanowskiales

Czekanowskiales, a characteristic gymnosperm group of Eurasia spanning the Late Triassic to Early Cretaceous, became extinct by the end of the Cretaceous. This order includes *Czekanowskia*, *Phoenicopsis*, *Sphenarion*, *Solenites*, *Leptostrobus*, and *Ixostrobus*. They are depicted as deciduous trees or shrubs possessing a short shoot, bearing scale leaves and fascicled leaves. Czekanowskiales had a wide distribution and are frequently regarded as representative of temperate and warm–temperate climates with seasonal variation. The fossil records reveal their ecological amplitude, spanning montane, lowland, and humid-to-semiarid paleohabitats across temperate–subtropical zones [38,39,40,51,52,53,54]. Czekanowskiales exhibit high generic and specific richness in the Baishan flora. Representatives, including *Czekanowskia*, *Phoenicopsis*, *Sphenarion*, and *Solenites,* have been identified, providing further evidence for a warm and humid temperate climate.

6. Coniferopsida

Coniferopsida constitute the dominant extant gymnosperm group, comprising 51 genera and over 550 species of trees or shrubs. They are primarily distributed in montane coniferous forests in temperate regions. Studies of fossil leaves and reproductive organs have confirmed the ecological adaptive continuity between the Mesozoic and extant Coniferopsida.

The Coniferopsida in the Baishan flora include *Pityocladus*, *Pityophyllum*, *Pityospermum*, *Schizolepis*, and *Scarburgia*, 5 genera (10 species) of Pinaceae. Additionally, it includes *Ferganiella*, *Lindleycladus*, and *Podozamites*, 3 genera (4 species) of Podozamitales. The compositional and morphological evidence indicates that Pinaceae is the largest Coniferopsida group in the Baishan flora. Analogous to extant Pinaceae trees or shrubs, these taxa represent dominant components of coniferous forests, primarily occurring in temperate to warm–temperate zones of the Northern Hemisphere, or in subtropical highlands with cool climates.

#### 2.2.3. Reconstruction of the Yingzuilazi Formation Plant Communities

The paleocommunities fundamentally align with the extant plant communities. However, the preservation of disarticulated plant organs constrains a paleobotanical reconstruction. Therefore, reconstructing paleocommunities based on fossil materials has become a critical focus of research. This approach holds substantial theoretical and practical value for understanding plant community assembly patterns in different geological periods, analyzing the patterns of environmental evolution, investigating coal-forming material sources, restoring the paleovegetation profiles, and elucidating the origins and development of modern plant communities. Furthermore, it enables direct visualization of paleolandscapes across geological periods and proves indispensable for interpreting paleobiotic habitats, restoring paleoenvironments, and reconstructing paleoecosystems.

**Figure 11:**
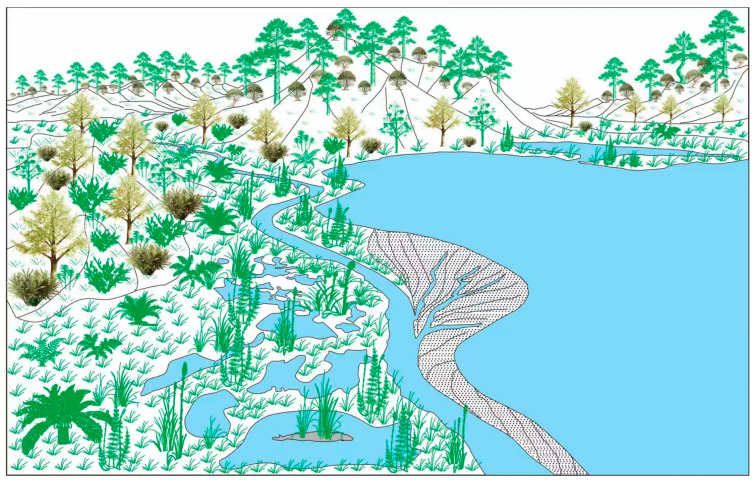
Paleoenvironmental reconstruction of the plant community.

We reconstructed four plant communities based on a systematic analysis of the compositional characteristics and paleoecological attributes of the Yingzuilazi Formation flora from the Baishan Basin, Jilin Province (Figure 11).

1. Riparian–Wetland Community

The riparian–wetland community occupied the understory or the humid marshes and floodplain zones adjacent to rivers and lakes. It exhibited a low diversity of flora, dominated solely by the order Equisetales: *Equisetites exiliformis* Sun et Zheng, *Equisetites longevaginatus* Wu, and *Equisetostachys* sp. This community exhibited low structural complexity, lacking well-defined vertical stratification, with *Equisetites* and *Equisetostachys* as the sole components. Such structural simplicity resembles modern herbaceous communities in lakeside marshes and waterlogged floodplains.

2. Lowland Community

The lowland community in the Baishan flora comprised Filicopsida and Cycadopsida. It primarily occupied gentle-sloping floodplains or subaerial deltas characterized by moist, well-drained soils, including *Todites* sp. (Osmundaceae, Filicales), *Coniopteris angustiloba* Brick (Dicksoniaceae), *Coniopteris* sp. (Dicksoniaceae), and *Gleichenites* sp. (Gleicheniaceae). This community also contained *Tyrmia* cf. *acrodonta* Wu, a Bennettitalean taxon.

Filicopsida exhibited higher diversity and represented the dominant taxa in this community, while Cycadopsida constituted the subordinate element. The reconstructed vegetation structure indicates a gentle topography with persistent moisture, proximal to slow-moving water bodies (e.g., rivers or lakes). Herbaceous ferns thrived, interspersed with scattered clumps of low-growing cycads.

3. Montane Slope Community

The montane slope community occupied elevated terrains within the basin or surrounding highlands. It was characterized by shrubs and deciduous mesophytic trees, with hygrophilous herbaceous plants (e.g., ferns) occurring sporadically in the lower slope margins or the understory. The dominant elements included Ginkgoopsida and Czekanowskiales, such as *Ginkgo huttoni* (Sternberg) Heer, *Ginkgoites sibiricus* (Heer) Seward, *Pseudotorellia* sp., *Baiera baishanensis* Zhao et Sun, *Baiera* sp., *Czekanowskia setacea* Heer, *Czekanowskia* sp., *Phoenicopsis* (*Culgoweria*) *uralensis* Kiritchkova, *Phoenicopsis* sp., *Solenites baishanensis* Li et Sun, *Solenites murrayana* Lindley et Hutton, *Solenites gracilis* Li et Sun, *Solenites* sp.1, *Solenites* sp.2, *Sphenarion* sp.1, *Sphenarion* sp.2, and *Ixostrobus delicatus*.

Czekanowskiales were the dominant taxa, with Ginkgoopsida as a constituent element. The vegetation was characterized by a broadleaved canopy of scattered tall Ginkgoopsida. The understory thrived with Czekanowskiales, which grew as shrubs or small trees bearing clusters of linear leaves on short shoots. The dimly lit understory, coupled with the relatively high humidity of both the air and soil, supported herbaceous growth, such as ferns. At higher elevations on the slope, Coniferopsida, such as Pityocladus and Pityophyllum, were present, forming a mixed coniferous–broadleaved forest belt together with Ginkgoopsida and Czekanowskiales.

4. Montane Highland Community

The montane highland community occupied the higher elevations to the near-summit zones of the mountains surrounding the basin. The vegetation was dominated by arboreal and shrubby Coniferopsida, including Coniferales taxa, such as *Pityocladus* sp.1, *Pityocladus* sp.2, *Pityophyllum staratschini* (Heer) Nathorst, *Pityophyllum lindstroemi* Nathorst, *Pityophyllum* sp.1, *Pityophyllum* sp.2, *Pityospermum* sp.1, *Pityospermum* sp.2, *Schizolepis moelleri* Seward, and *Scarburgia hillii* Harris, and Podozamitales taxa, including *Ferganiella podozamioides* Lih, *Ferganiella* sp., *Lindleycladus* sp., and *Podozamites* spp.

This community exhibited a Coniferopsida-dominated vegetation structure with arboreal and shrubby growth forms. The higher elevation reduced humidity of air and soil, and herbaceous development was limited, resulting in scarcity of such elements within the community.

### 2.3. Foliar Physiognomic Paleoclimatic Analysis of the Flora

Plant growth processes exhibit coordinated unity with environmental factors, with the climate serving as a critical determinant of plant ecological structure and development. Distinct vegetation types exist under different climates [55]. Leaves indicate the specific growth environments; some scholars assert that the morphological traits of plant leaves offer significant insights into environmental change. As such, the examination of leaf physiognomy is instrumental in the analysis of paleoclimate and the inference of paleoenvironmental conditions [56,57,58,59]. Foliar physiognomy encompasses the collective macroscopic leaf characteristics. Dolph et al. [60] categorized foliar physiognomy into eight traits: leaf margin type, drip tips, leaf size class, leaf shape, venation pattern, leaf texture, leaf base morphology, and vein density. These traits reflect plant ecological adaptations and sensitively indicate the climate. This study statistically analyzed the foliar physiognomic traits in the Baishan flora. Applying the actualistic principle, we interpret these features to infer the paleoclimate. Notably, Sphenopsida were excluded from the analysis, as their leaf traits primarily reflect phylogenetic constraints rather than environmental influences.

#### 2.3.1. Leaf Size Class Analysis

The leaf size class refers to categorical divisions of leaf area. Raunkiaer [61] proposed that the distribution of leaf size classes can serve as an indicator of climate. His classification comprises six grades: leptophyll (<25 mm^2^), nanophyll (25–225 mm^2^), microphyll (225–2025 mm^2^), mesophyll (2025–18,225 mm^2^), macrophyll (18,225–164,025 mm^2^), and megaphyll (>164,025 mm^2^) [31].

We quantified the leaf size classes in the Baishan flora and compared them with modern vegetation under varying climates (Table 5). The data reveal a general pattern where tropical regions exhibit the highest proportion of large-leaved species. This percentage decreases with increasing latitude, while small-leaved species become more prevalent. Consequently, the leaf size correlates strongly with climate. In the Baishan flora, microphylls dominated (40.1%), followed by mesophylls (28.6%), nanophylls (13.0%), macrophylls (11.2%), and leptophylls (7.1%). Comparing these with modern climatic zone vegetation and Mesozoic floras (Baojishan Yaojie Formation), the leaf size class distribution of the Baishan flora aligns with warm–temperate to temperate floras, leaning toward temperate conditions. This pattern suggests a warm–humid temperate climate.

#### 2.3.2. Leaf Margin Analysis

**Figure 12:**
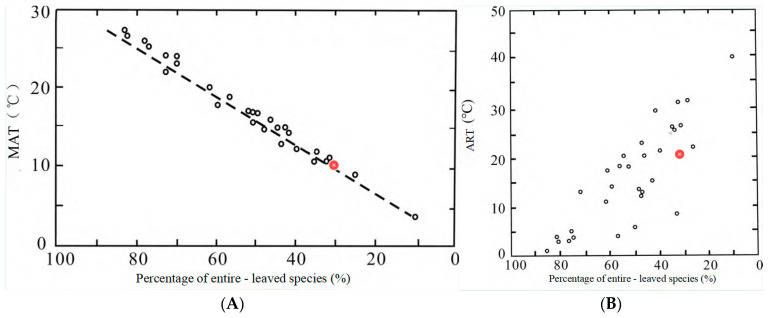
Relationship between the percentage of entire-margined leaves and MAT/ATR [64]. (**A**) MAT of the Early Cretaceous Baishan Basin, (**B**) ATR of the Early Cretaceous Baishan Basin.

The percentage of entire-margined species within a flora is used to infer the paleoclimate and the approximate mean annual temperature (MAT) and annual temperature range (ATR). Wolfe [64] demonstrated that the proportion plants with entire-margined leaves decreases progressively with declining temperatures (increasing latitude). In the Baishan flora, plants with entire-margined leaves account for 30.4%, while dissected-margined plants account for 69.6%. This distribution indicates a warm–humid temperate climate (Table 6). Wolfe’s [64] regressions establish that a higher MAT corresponds to increased entire-margined species percentages, whereas a greater ATR reduces this proportion (Figure 12). Applying Wolfe’s model to our data yields an estimated MAT of 10 °C and ATR of 21 °C for the Early Cretaceous Baishan Basin, consistent with warm–humid temperate conditions.

Synthesizing multiple foliar physiognomic analyses indicates warm–humid temperate climatic conditions for the Baishan flora. The application of leaf margin–climate correlations provide approximate estimates of a 10 °C mean annual temperature and 21 °C annual temperature range. These quantitative reconstructions align with the paleoclimatic inferences derived from the floristic composition characteristics.

## 3. Discussion

The Earth was characterized by a greenhouse climate devoid of permanent ice sheets during the Cretaceous period. In East Asia, the influence of the Paleo-Pacific Ocean may have given rise to an incipient monsoon system, resulting in overall warm and humid conditions that fostered the proliferation of flora and fauna and facilitated the development of lacustrine basins. The Baishan Basin is an Early Cretaceous small-scale faulted continental sedimentary basin. Its tectonic setting is situated at the northeastern margin of the North China Craton, bounded on the east by the Tan–Lu Fault Zone, on the north by the Tianshan–Xingmeng Orogenic Belt, on the south by the Liaodong Block, and extending eastward to connect with the basement of the Korean Peninsula. Climatically, it belonged to a warm and humid temperate zone. The stratigraphic sequence within the basin, in ascending order, comprises the Yingzuilazi, Linzitou, and Shiren Formations. The Yingzuilazi Formation is distributed in the northern part of the Baishan Basin along a north–northeast trend and consists of a coal-bearing series dominated by lacustrine deposits with intercalated fluvial and swamp facies. The lower part of the formation is characterized by purplish, thick-bedded conglomerates interbedded with medium-bedded siltstones, representing fluvial channel bar or lakeshore deposits, which reflect the relatively arid climatic conditions during the initial depositional stage of the Yingzuilazi Formation. The middle part consists mainly of gray, yellow, or yellowish-green, medium- to thin-bedded sandstones, siltstones, and mudstones, with well-developed horizontal bedding, indicating deposition in a shallow lacustrine environment. The upper part is dominated by gray and yellow, medium- to thick-bedded sandstones intercalated with siltstones and mudstones, representing a gentle shallow littoral-to-nearshore lacustrine setting. Based on the lithological color characteristics, both the middle and lower parts of the Yingzuilazi Formation are interpreted to have been deposited under warm and humid paleoenvironmental conditions. The Yingzuilazi Formation flora belongs to the Early Cretaceous Northern Chinese Floristic Province or the Siberian–Canadian Floristic Realm, constituting a significant component of warm–temperate to temperate floras.

The Yingzuilazi Formation flora in Baishan City, Jilin, is dominated by gymnosperms and ferns. Among the gymnosperms, Coniferopsida exhibit the highest diversity and widest distribution, with *Pityophyllum* as the dominant genus typically inhabiting temperate to warm–temperate zones or subtropical montane slopes or highlands with cool climates. Ginkgoaleans, primarily represented by Ginkgoales, favor well-drained slopes in tropical, subtropical, and warm–temperate regions. Czekanowskiales thrive in warm semiarid climates with seasonal precipitation or a cooler climate, requiring well-drained soils. Filicopsida show the greatest richness in Dicksoniaceae, occurring across tropical to temperate zones. Sphenopsids, represented by *Equisetites* and *Equisetostachys,* adapted to diverse Mesozoic climates but exhibited a preference for temperate regimes. Cycadopsida and Gnetophyta are rare, with occasional occurrences of *Tyrmia* cf. *acrodonta* Wu, *Ephedra* sp., and *Ephedrites* sp. Based on an analysis of the floral composition, it can be inferred that the climate belonged to the warm–temperate to temperate coastal monsoon climate zone of northern China, under the influence of marine monsoons from the Palaeo-Pacific, during the depositional period of the Early Cretaceous Yingzuilazi Formation in the Baishan Basin.

Palynomorphs from the Yingzuilazi Formation consist predominantly of fern spores and gymnosperm pollen. According to a previous study, this palynological assemblage has been provisionally classified as the *Cicatricosisporites*–*Abietineaepollenites* [28]. Evidently, temperate and warm–temperate elements dominate both the macrofloral and palynological records, while the arid-adapted Gnetophyta and tropical Cycadopsida are exceptionally scarce.

An integrated analysis of floristic composition, foliar physiognomic statistics, and palynofloral characteristics indicates seasonal climatic variability during the Yingzuilazi Formation deposition. Warm–humid rainy seasons supported vigorous vegetation growth, while dry seasons or drought years scattered xerophytic plants. Thus, the evidence supports warm–humid temperate conditions in the Early Cretaceous Baishan Basin. The warm and humid climatic conditions inferred from the regional geological analysis of the Baishan Basin are consistent with those indicated by the Yingzuilazi Formation flora.

## 4. Conclusions

The Early Cretaceous Yingzuilazi Formation flora in Baishan City, Jilin, constitutes a Mesozoic assemblage dominated by gymnosperms and ferns. It includes Sphenopsida (6.52%), Filicopsida (8.70%), Cycadopsida (2.17%), Ginkgoopsida (10.87%), Czekanowskiales (26.09%), Coniferopsida (30.43%), Gnetophyta (4.35%), and gymnosperm reproductive structures (10.87%). The total diversity reaches 27 genera and 46 species. The floristic composition shows a close affinity to the Yixian Formation flora of western Liaoning, establishing this locality as the easternmost distribution of the Jehol Biota in China. This assemblage offers critical stratigraphic evidence for interpreting Jehol flora diversity, paleoecological patterns, and paleoenvironmental proxies.

The taphonomic analysis reveals predominantly autochthonous to parautochthonous preservation. By integrating paleoecological traits of fossil taxa with habitats of extant relatives, we reconstruct four Early Cretaceous plant communities. These are the riparian–wetland communities dominated by Sphenopsida, lowland communities primarily consisting of Filicopsida and Cycadopsida, montane slope communities where Ginkgoopsida and Czekanowskiales prevail, and montane highland communities characterized by drought and cold-tolerant Coniferopsida. These reconstructions elucidate the topographic controls on vegetation distribution in the Baishan Basin and deliver new paleoecological constraints for evaluating habitat heterogeneity within the Jehol Biota.

Discoveries and studies of the Jehol Biota in Baishan City provide crucial fossil evidence for understanding Early Cretaceous terrestrial ecosystem biodiversity and evolutionary radiation. They further enhance knowledge regarding the biota’s geographic and stratigraphic distribution patterns.

The warm–temperate humid climate reconstructed for the Baishan Basin serves as a deep-time analogue for predicting vegetation shifts under current global warming scenarios, particularly in mid-latitude East Asia. The riparian–wetland communities rich in aquatic plants suggest lacustrine depositional settings in the Baishan Basin, potentially correlating with organic-rich shales suitable for unconventional hydrocarbon exploration.

## Figures and Tables

**Table 1:** Stratigraphy and fossil distribution in the Yingzuilazi Formation.

Stratigraphic Unit	Lithology	Interpreted Environment	Fossils
19	Yellow Feldspathic Quartz Sandstone	Shallow Lake Near-shore	
18	Greyish-Green Siltstone	Shallow Lake	
17	Yellow–Green Silty Mudstone Interbedded with Thin Grey Fine Sandstone	Gentle Shallow Lake Nearshore	Bivalves
16	Grey Medium-Thick-Bedded Fine Sand-stone with Thin Siltstone		Abundant Bivalves
15	Grey–Black Siltstone	Shallow Lake	
14	Yellow–Green Argillaceous Siltstone		
13	Grey–Green Fine Sandstone		
12	Yellow Sandstone with Thin Grey Argilla-ceous Siltstone		Ostracods
11	Grey Medium-Thin-Bedded Silty Mudstone		
10	Yellow–Green, Yellow Silty Shale		Plant Fossils
9	Light Grey, Grey Medium-Thick-Bedded Sandstone and Mudstone Interbeds		
8	Grey Medium-Bedded Mudstone and Silty Mudstone Interbeds		Plants, Conchostracans, Bi-valves, Gastropods, Insects, Fishes, and Caudate Am-phibian
7	Grey Mudstone with Grey–White Argilla-ceous Siltstone		
6	Grey–Green Silty Mudstone		
5	Yellow with Green Argillaceous Siltstone		
4	Dark Purple Pebbly Siltstone	Braided River/Alluvial Plain	Indicative of Arid Climate
3	Dark Purple Siltstone	Braided River/Alluvial Plain	
2	Purple Pebbly Sandstone	Braided Channel	

**Table 2:** Plant fossil composition of the Yingzuilazi Formation in Baishan, Jilin Province.

Class	Genus	Species	Percentage (%)
Sphenopsida	*Equisetites*	*Equisetites* cf. *exiliformis* Sun et Zheng; *Equisetites longevaginatus* Wu	4.3
	*Equisetostachys*	*Equisetostachys* sp.	2.2
Filicopsida	*Todites*	*Todites* sp.	2.2
	*Coniopteris*	*Coniopteris angustiloba* Brick; *Coniopteris* sp.	4.3
	*Gleichenites*	*Gleichenites* sp.	2.2
Cycadopsida	*Tyrmia*	*Tyrmia* cf. *acrodonta* Wu	2.2
Ginkgoopsida	*Ginkgo*	*Ginkgo huttoni* (Sternberg) Heer	2.2
	*Ginkgoites*	*Ginkgoites sibiricus* (Heer) Seward	2.2
	*Pseudotorellia*	*Pseudotorellia* sp.	2.2
	*Baiera*	*Baiera baishanensis* Zhao et Sun; *Baiera* sp.	4.3
Czekanowskiales	*Czekanowskia*	*Czekanowskia setacea* Heer; *Czekanowskiales* sp.	4.3
	*Phoenicopsis*	*Phoenicopsis* sp.; *Phoenicopsis* (*Culgoweria*) *uralensis* Kiritchkova	4.3
	*Solenites*	*Solenites baishanensis* Li et Sun; *Solenites murrayana* Lindley et Hutton; *Solenites gracilis* Li et Sun; *Solenites* sp.1; *Solenites* sp.2	10.9
	*Sphenarion*	*Sphenarion* sp.1; *Sphenarion* sp.2	4.3
	*Ixostrobus*	*Ixostrobus delicatus* Sun et Zheng	2.2
Coniferopsida	*Pityocladus*	*Pityocladus* sp.1; *Pityocladus* sp.2	4.3
	*Pityophyllum*	*Pityophyllum staratschini* (Heer) Nathorst; *Pityophyllum lindstroemi* Nathorst; *Pityophyllum* sp.1; *Pityophyllum* sp.2	8.7
	*Pityospermum*	*Pityospermum* sp.1; *Pityospermum* sp.2	4.3
	*Schizolepis*	*Schizolepis moelleri* Seward	2.2
	*Scarburgia*	*Scarburgia hillii* Harris	2.2
	*Ferganiella*	*Ferganiella podozamioides* Lih; *Ferganiella* sp.	4.3
	*Lindleycladus*	*Lindleycladus* sp.	2.2
	*Podozamites*	*Podozamites* spp.	2.2
Gentopsida	*Ephedra*	*Ephedra* sp.	2.2
	*Ephedrites*	*Ephedrites* sp.	2.2
Fructus et Semina	*Strobilites*	*Strobilites* sp.1; *Strobilites* sp.2	4.3
	*Carpolithus*	*Carpolithus* sp.1; *Carpolithus* sp.2; *Carpolithus* spp.	6.5

**Table 3:** Standard table forjudging taphonomic types of fossil plants [33].

	Burial Type	Autochthonous/Parautochthonous	Allochthonous
Criterion			
Preservation State		Well preserved; possess rhizomes, stoloniferous bases, root remnants, or rhizomorphic structures; leaf outlines distinct and venation patterns clearly identifiable.	Highly fragmented with low completeness.
Sorting Condition		Variable size and diverse taxa.	Uniform size with mass accumulation.
Orientation Pattern		No preferred orientation.	Exhibits preferred orientation.
Organ Resist		Thin-textured leaves and strobiloid reproductive organs.	Well-preserved coriaceous leaves.

**Table 4:** Taphonomic characteristics of Early Cretaceous fossil plants in Baishan, Jilin Province.

Species	Stratum	Host Rock	Abundance	Preservation State	Burial Style
*Equisetites* cf. *exiliformis* Sun et Zheng	K_1_*y*	gray–yellow sandstone	++	leaf sheaths intact, possess sclerified ridges, distinct longitudinal grooves	autochthonous
*Equisetites longevaginatus* Wu	K_1_*y*	gray–yellow sandstone	++	leaf sheaths intact, possess sclerified ridges, distinct longitudinal grooves	autochthonous
*Equisetostachys* sp.	K_1_*y*	gray–yellow sandstone	+	strobili with small scutella, basal stalks connected to stems	autochthonous
*Coniopteris angustiloba* Brick	K_1_*y*	gray–yellow sandstone	+++	bipinnate fronds, clear venation, rachises showing distinct longitudinal striations	autochthonous
*Coniopteris* sp.	K_1_*y*	gray–yellow sandstone	++	bipinnate fronds, clear venation, rachises showing distinct longitudinal striations	autochthonous
*Tyrmia* cf. *acrodonta* Wu	K_1_*y*	gray siltstone	+	pinnae attached to rachis, lobes largely intact, clear venation	parautochthonous
*Ginkgo huttoni* (*Sternberg*) Heer	K_1_*y*	gray mudstone	+++	leaves intact, clear venation	autochthonous
*Ginkgoites sibiricus* (Heer) Seward	K_1_*y*	gray–yellow siltstone	++	leaves intact, clear venation	autochthonous
*Pseudotorellia* sp.	K_1_*y*	gray–yellow sandstone	++	leaves largely intact, clear venation	parautochthonous
*Baiera baishanensis* Zhao et Sun	K_1_*y*	gray–yellow siltstone	+++	leaves intact, clear venation	autochthonous
*Baiera* sp.	K_1_*y*	gray–yellow sandstone	+	leaves largely intact, venation indistinct	parautochthonous
*Czekanowskia setacea* Heer	K_1_*y*	gray mudstone	++	leaves largely intact, venation indistinct	parautochthonous
*Czekanowskiales* sp.	K_1_*y*	gray–yellow siltstone	+	leaves largely intact, clear venation	autochthonous/parautochthonous
*Phoenicopsis* sp.	K_1_*y*	yellow siltstone	+++	leaves largely intact, clear venation	autochthonous
*Phoenicopsis* (*Culgoweria*) *uralensis* Kiritchkova	K_1_*y*	yellow–green mudstone	+++	linear leaves clustered on short shoots, leaves complete, clear venation	autochthonous
*Solenites baishanensis* Li et Sun	K_1_*y*	yellow siltstone	+++	linear leaves clustered on short shoots, leaves complete, clear venation	autochthonous
*Solenites murrayana* Lindley et Hutton	K_1_*y*	yellow–green sandstone	+++	leaves largely intact, venation indistinct	autochthonous
*Solenites gracilis* Li et Sun	K_1_*y*	gray–yellow mudstone	+++	short shoots present, leaves complete, clear venation	autochthonous
*Solenites* sp.1	K_1_*y*	yellow–green siltstone	+++	short shoots present, leaves complete, clear venation	autochthonous
*Sphenarion* sp.1	K_1_*y*	gray–yellow mudstone	+	leaves largely intact, clear venation	parautochthonous
*Ixostrobus delicatus* Sun et Zheng	K_1_*y*	yellow–green siltstone	++	structures complete	autochthonous
*Pityocladus* sp.1	K_1_*y*	yellow–green siltstone	+	structures largely complete	parautochthonous
*Pityophyllum staratschini* (Heer) Nathorst	K_1_*y*	yellow–green sandstone	+++	structures complete	autochthonous
*Pityophyllum lindstroemi* Nathorst	K_1_*y*	yellow sandstone	+++	leaves intact, clear venation	autochthonous
*Pityophyllum* sp.1	K_1_*y*	yellow–green sandstone	+++	leaves intact, clear venation	autochthonous
*Pityospermum* sp.1	K_1_*y*	yellow–green sandstone	+	structures complete	autochthonous/parautochthonous
*Schizolepis moelleri* Seward	K_1_*y*	gray mudstone	++	structures complete	autochthonous
*Scarburgia hillii* Harris	K_1_*y*	gray–yellow sandstone	+	structures complete	autochthonous/parautochthonous
*Ferganiella podozamioides* Lih	K_1_*y*	gray–yellow siltstone	++	leaves complete, dichotomous branching, clear venation	autochthonous
*Ferganiella* sp.	K_1_*y*	gray sandstone	+++	leaves intact, clear venation	autochthonous
*Lindleycladus* sp.	K_1_*y*	gray–yellow mudstone	+++	leaves intact, clear venation	autochthonous
*Ephedra* sp.	K_1_*y*	yellow–green sandstone	+	structures complete	autochthonous/ parautochthonous
*Strobilites* sp.1	K_1_*y*	gray sandstone	++	structures largely complete	autochthonous
*Carpolithus* sp.1	K_1_*y*	gray–yellow sandstone	+++	structures complete	autochthonous

Abundance is categorized as: “+” for rare, “++” for abundant, and “+++” for rich

**Table 5:** Comparison of leaf size among the studied flora, the fossil plants of Yaojie Formation in Baojishan Basin, and modern vegetation.

Community Type (Location)	Leptophyll	Nanophyll	Microphyll	Mesophyll	Macrophyll	Megaphyll
Tropical Rainforest (Africa, Eastern Ecuador) ^1^	0	0	9	64	27	0
Tropical Rainforest (Brazil, 1°27′ S) ^1^	2.3	3.2	15.1	68.3	11.0	0
Temperate Rainforest (Brazil, Caioba 26° S) ^1^	0	8.8	14.4	64.4	11.1	1.1
Evergreen Broadleaved Forest (Zhejiang, China) ^1^	0	4.1	53.3	37.1	5.4	0
Evergreen Broadleaved Forest (Lushan, China, 29°35′ N) ^1^	0	7.0	52.9	39.7	0.4	0
Baojishan Yaojie Formation Flora (Gansu) ^2^	7.8	13.3	40.7	25.0	15.2	0
Temperate Montane Coniferous Forest (Changbai Mountains) ^1^	6.5	13.0	39.5	31.8	8.8	0
Baishan Yingzuilazi Formation Flora	7.1	13.0	40.1	28.6	11.2	0

^1^ Data for modern vegetation from Wu, J.H., *Plant Geography, 4th ed.* [62]; ^2^ Liu, S.X. et al., 2021 [63].

**Table 6:** Relationship between the percentage of floras with whole leaves and climate zone.

	Flora or Vegetation Type	Entire Margins (%)	Climatic Zone
Fossil Floras	Xinjiang Altay Fossil Flora (Paleocene) ^3^	77	Tropical
	Bear Den Member (Late Eocene) ^1^	33	Warm–Temperate
	Baishan Early Cretaceous Flora	30.4	Temperate
	Gansu Baojishan Flora (Middle Jurassic) ^4^	35.4	Warm–Temperate
	Chalk Bluffs (Middle Eocene) ^1^	46	Orizaban Subtropical
	Wilcox (Eocene) ^1^	83	Tropical
Modern Vegetation	Pennsylvania ^1^	30	Temperate
	Evergreen Broadleaf Forest (Zhejiang) ^2^	46	Subtropical
	Taiwan (0–500 m elev.) ^1^	61	Subtropical
	Philippines ^1^	76	Tropical
	Panama Lowland Rainforest ^1^	88	Tropical

^1^ Date from Hickey, 1977 [65]; ^2,3^ Date from Li Bai, 1985 [66]; ^4^ Date from Liu, 2021 [67].

## Data Availability

The original contributions presented in the study are included in the article, further inquiries can be directed to the corresponding author.
